# Combined Overlap Extension PCR Method for Improved Site Directed Mutagenesis

**DOI:** 10.1155/2016/8041532

**Published:** 2016-11-22

**Authors:** Hasnain Hussain, Nikson Fatt-Ming Chong

**Affiliations:** Department of Molecular Biology, Faculty of Resource Science and Technology, Universiti Malaysia Sarawak, 94300 Kota Samarahan, Sarawak, Malaysia

## Abstract

The combined overlap extension PCR (COE-PCR) method developed in this work combines the strengths of the overlap extension PCR (OE-PCR) method with the speed and ease of the asymmetrical overlap extension (AOE-PCR) method. This combined method allows up to 6 base pairs to be mutated at a time and requires a total of 40–45 PCR cycles. A total of eight mutagenesis experiments were successfully carried out, with each experiment mutating between two to six base pairs. Up to four adjacent codons were changed in a single experiment. This method is especially useful for codon optimization, where doublet or triplet rare codons can be changed using a single mutagenic primer set, in a single experiment.

## 1. Introduction

Site directed mutagenesis is a technique used for substitution, addition, and deletion of specific base sequences in DNA [1]. It is an important tool to generate mutants with altered amino acid sequences for enzyme studies, investigation of the relationship between structure and functions of proteins, and functional analysis of genes or their regulatory sequences [2–4]. Altering the amino acid sequence of an enzyme has been used to improve enzyme properties such as catalytic activity, thermostability, and chemical tolerance [5, 6]. Furthermore, site directed mutagenesis is also used in codon optimization to remedy codon bias during heterologous expression of proteins [7, 8].

PCR based mutagenesis methods are advantageous because they are rapid and have very high mutation efficiencies [3]. Mutations are introduced through mutagenic primers which contain one or more mismatched bases [1, 2]. These mutagenic primers are incorporated during PCR and the mutant DNA is amplified exponentially [1, 2].

Among the PCR based methods, the overlap extension PCR (OE-PCR) and asymmetrical overlap extension PCR (AOE-PCR) are notable for their simplicity and efficiency in multiple-site mutagenesis [2, 9]. The OE-PCR method consists of two primary PCR reactions which generate mutant DNA fragments with overlapping ends and a secondary reaction which joins the two fragments into a single fragment [9]. The OE-PCR method is both durable and robust [10].

The AOE-PCR method consists of two primary PCR reactions which generate single stranded mutant DNA with overlapping ends and an incubation step where the overlapping single stranded DNA anneal together and are extended to produce a single PCR product [2]. In the AOE-PCR method, excess gene specific primers but limited mutagenic primers are used, resulting in the formation of single stranded DNA. Compared to the OE-PCR method, it is faster as it bypasses gel purification of the primary PCR products and simplifies the secondary PCR reaction into a single incubation step [2].

In this work, the combined overlap extension PCR (COE-PCR) method, which combines the advantages of the OE-PCR and AOE-PCR methods, was used for codon optimization of the* Solanum tuberosum* isoamylase* Stisa2* gene for heterologous expression in* E. coli*. This method is reliable, robust, and faster than conventional OE-PCR methods.

## 2. Materials and Methods

### 2.1. Primer Design

The gene specific primers (GSP): forward (FGSP) and reverse (RGSP), were designed to contain suitable restriction sites and flank the forward mutagenic primers (FMP) and reverse mutagenic primers (RMP). The mutagenic primers (MP) were complementary to each other, with the mutagenic bases in the center of each primer, and were between 17 and 32 bp in length depending on the number of base pairs to be changed. The mutagenic primers were chosen to have the same annealing temperature so that both primary PCR reactions could be performed simultaneously. The mutagenic primers were designed with the aid of PrimerX program (http://www.bioinformatics.org/primerx/index.htm) and checked for primer-dimer and hairpin formation using OligoAnalyzer 3.1 (http://sg.idtdna.com/calc/analyzer). All primers were synthesized and trityl-on-purified (TOP) by a commercial vendor. The list of primers is shown in Table 1.

### 2.2. The COE-PCR Method

The COE-PCR method consists of two primary PCR reactions and a secondary PCR reaction. The primary PCR reactions follow a standard PCR reaction but utilize limited mutagenic primers. The primary PCR reaction for each primer pair was carried out in separate tubes, using 5 pmol GSP, 1.25 pmol MP, 2 U* Pfu* DNA polymerase (Fermentas), 5 nmol dNTP mix, 62.5 nmol MgCl_2_, and 2.5 pg template DNA in a 25 *μ*L reaction. The template was the* Stisa2* gene (Accession: AY132997) in a pSTAG vector [11, 12]. The PCR thermocycling profile used was as follows: initial denaturation for 5 minutes at 95°C, followed by 30 cycles of 30 seconds at 94°C, 30 seconds at annealing temperature, and 2–4 minutes at 72°C. This was followed by a final elongation of 72°C for 5 minutes. *T*_*a*_ was set at 50–55°C for all primer sets to match *T*_*a*_ of the gene specific primers rather than *T*_*m*_ calculated by PrimerX program.

The primary PCR products were visualized on 1% agarose gel stained with ethidium bromide. The amount of DNA ladder (Fermentas Gene Ruler 1 kb DNA ladder) loaded was standardized at 200 ng and 300 ng for small- and medium-sized lanes, respectively. PCR products loaded were standardized at five microliters upon completion of PCR cycles. After the primary PCR, if a single band of the expected size was obtained, gel purification was unnecessary. If multiple bands were obtained, the band of the correct expected size was excised and gel-purified. The concentration of the PCR products was estimated by comparing band intensity with the DNA ladder using ImageJ software (http://imagej.nih.gov/ij/) [13].

**Figure 1:**
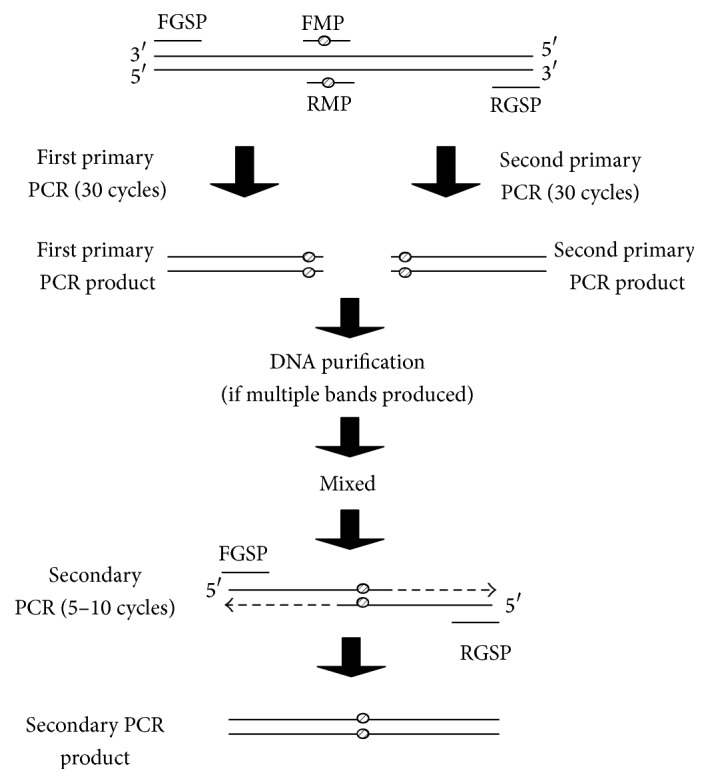
Overview of the combined overlap extension PCR (COE-PCR) method. The primary PCR reactions follow a standard PCR reaction, but with limited mutagenic primers. The primary PCR reactions produce double stranded mutagenic DNA fragments which have overlapping ends. In the secondary PCR reaction, the products from the primary PCR reaction are mixed in a 1 : 1 ratio and amplified in a modified PCR reaction consisting of 5–10 cycles to produce full length double stranded mutagenic DNA (FGSP: forward gene specific primer; RGSP: reverse gene specific primer; FMP: forward mutagenic primer; RMP: reverse mutagenic primer).

In the secondary PCR reaction, 200–400 ng of each primary PCR product was used as template; 4 pmol FGSP, 4 pmol RGSP, 1 U* Pfu* DNA polymerase, 8 nmol dNTP mix, and 100 nmol MgCl_2_ were used in a single 40 *μ*L reaction. The PCR mixture was subjected to 5–10 PCR cycles, which produced the full length mutagenic DNA which was then excised and gel-purified to be used for cloning or downstream applications. An overview of the COE-PCR method is shown in Figure 1.

Table 2 shows the primer sets used for each mutagenesis experiment. The experiments were performed sequentially where the previous PCR product was used as the template in the subsequent PCR reaction. After the final mutation, the PCR product was purified, digested with restriction enzymes, and cloned into a pSTAG vector for sequencing purposes.

## 3. Results and Discussion

The COE-PCR method was successful in producing the desired mutations. DNA sequencing showed that all the point mutations were correctly performed.

The results of the primary PCR reactions depended mainly on the primer pair (GSP + MP), which determined whether optimization and gel purification were necessary. Most reactions were straightforward and did not require gel purification. However, several primary PCR reactions produced multiple bands including the expected band, which was gel excised. Meanwhile, only two primary PCR reactions required optimization before the expected band was obtained. Following the primary PCR reactions, the secondary PCR reactions were easily performed and none of the reactions required any optimization. A summary of the results is shown in Table 3.

**Figure 2:**
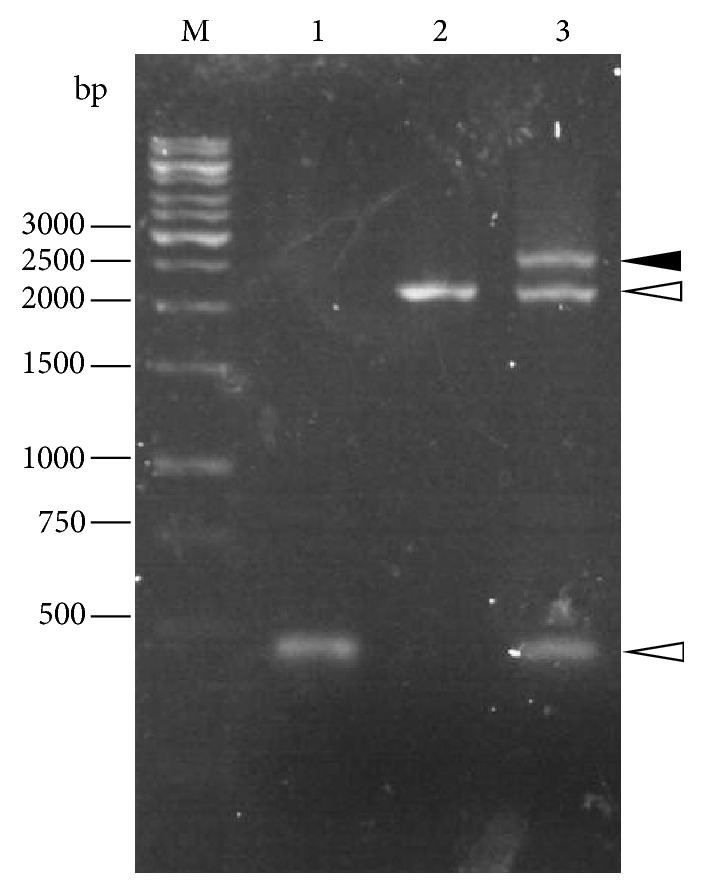
Results of COE-PCR for the 8th primer set. Both primary PCR reactions (lanes 1 and 2) produced single bands estimated at 450 bp and 2250 bp (indicated by white arrows), which matched the expected size. Since single bands were obtained, gel purification was unnecessary. The secondary PCR reaction (lane 3) produced a single band estimated at 2.7 kb (indicated by black arrow), which matched the expected size.

**Figure 3:**
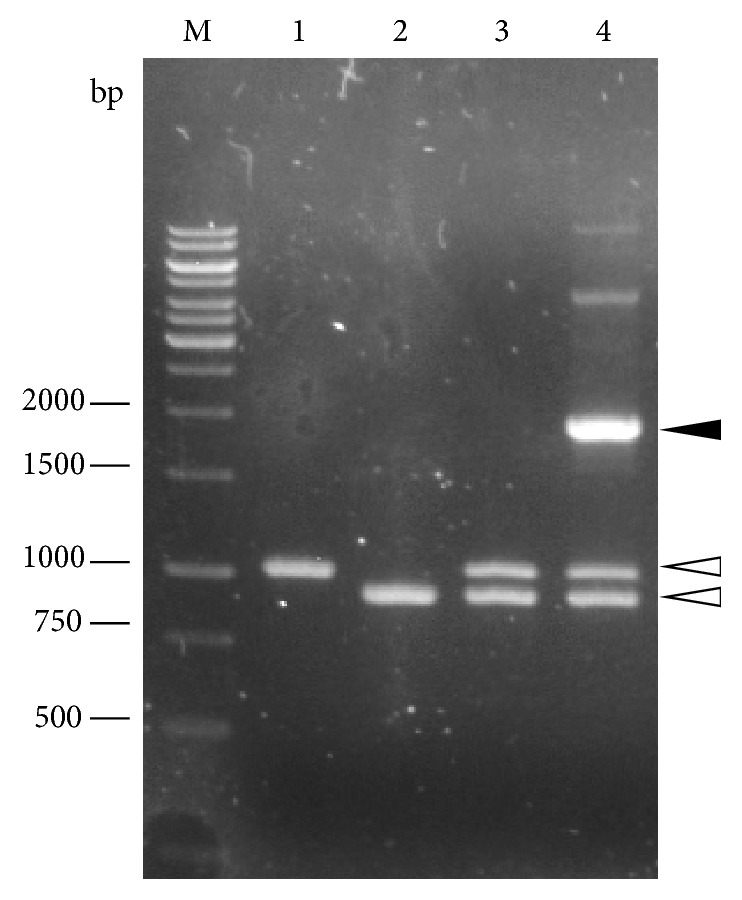
Results of COE-PCR for the 4th primer set. Both primary PCR reactions (lanes 1 and 2) produced single bands estimated at 1050 bp and 900 bp (indicated by white arrows), which matched the expected size and did not require gel purification. Lane 3 shows the results of the secondary PCR reaction after a single cycle, indicating that a single cycle was insufficient to amplify sufficient DNA. The secondary PCR reaction (lane 4) produced a bright band estimated at 1.9 kb (indicated by black arrow), which matched the expected size, and two additional bands of higher molecular weight. Since the 1.9 kb band matched the expected size, it was carefully excised, purified, and used as the template for the next mutagenesis experiment.

**Figure 4:**
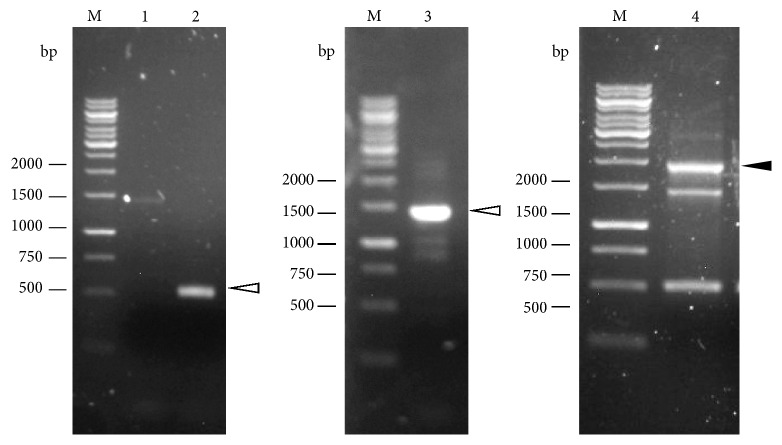
Results of COE-PCR for the 6th primer set. The first primary PCR reaction required optimization before producing the desired band in sufficient quantity. Before optimization (lane 1), the PCR reaction produced a faint band estimated at 1.4 kb, which matched the expected size. After optimization (lane 3), PCR produced multiple bands, with a bright band estimated at 1.4 kb (indicated by white arrow), which matched the expected size. Thus, the 1.4 kb band was excised and purified. The second primary PCR reaction (lane 2) produced a single band estimated at 500 bp (indicated by white arrow), which matched the expected size. The secondary PCR reaction (lane 4) produced a bright band estimated at 1.9 kb (indicated by black arrow), which matched the expected size.

Example results for three primer sets to describe the COE-PCR method are given as follows. The results of the 8th primer set showed the best case scenario where both the primary reactions produced single bands and the secondary reaction produced a single product of the expected size (Figure 2). The results of the 4th primer set showed straightforward primary reactions but a nonoptimum secondary reaction (Figure 3). The secondary PCR reaction yielded multiple bands, including a bright band which matched the expected size. This target band was excised and gel-purified. The results of the 6th primer set showed a requirement for optimization of the first primary PCR reaction (Figure 4) in order to produce the desired band. The secondary PCR reaction proceeded as expected.

The changes made to the OE-PCR method are minor, namely, the primer concentration used and the amount of the template DNA used in the secondary PCR reaction. However, this simple modification results in a method that combines the robustness of the OE-PCR with the speed and ease of the AOE-PCR methods. The COE-PCR can effectively change up to six base pairs at a time.

Similar to AOE-PCR, the use of asymmetrical primers causes the exhaustion of mutagenic primers after the primary reactions. Exhaustion of mutagenic primers is important to prevent residual mutagenic primers from competing with the overlapping ends, decreasing the efficiency of the secondary reaction [2]. Thus, double stranded DNA which is the primary PCR product need not be gel-purified if a single band is obtained. However, if the concentration of mutagenic primers is too low, the amount of primary PCR product would be reduced. Therefore, the concentration of mutagenic primers needs to be sufficient for amplification of the primary PCR products but should also be exhausted at the end of the PCR reaction. Although fast and easy, the AOE-PCR method as described [2, 14] was found to be unsuitable for larger base pair changes because the single stranded DNA produced had the tendency to anneal nonspecifically to other sections of DNA, producing multiple bands (unpublished work).

A novel feature of the COE-PCR method is the large amount of starting template in the secondary reaction. This allows sufficient amplification of mutant DNA in as few as 5–10 cycles. This is faster than the secondary PCR reaction of another improved OE-PCR method [15], which requires 30 cycles. The template in the secondary PCR reaction can be prepared simply by estimating the DNA concentration and mixing the PCR products in an equal ratio. DNA concentration need not be determined exactly but can be estimated simply by comparing band intensity to that of the DNA ladder using freely available software such as ImageJ software.

An important step in the COE-PCR method is size estimation of the PCR products. Correctly identifying the correct PCR products eliminates the need for optimization even if multiple bands are produced. While a single primary PCR product is ideal, sometimes, multiple bands are obtained. In this case it is easier to excise and purify the band of the correct estimated size than to optimize the experiment until a single band is obtained.

Errors introduced during PCR usually result from the DNA polymerase mediated synthesis or from the incorporation of missynthesized oligonucleotides [7]. In this work, all the point mutations were successfully performed using the eight primer sets, with no other mutation occurring elsewhere in the gene. This could be attributed to the use of high fidelity DNA polymerase and high quality TOP primers.

The COE-PCR method is highly suitable for site directed mutagenesis work where multiple adjacent base pairs are to be changed, such as in codon optimization for heterologous expression in* E. coli. *Replacing clusters of rare codons which inhibit protein expression in* E. coli* has been shown to improve protein expression [8, 16]. Using the COE-PCR method, changes of up to four adjacent codons were performed using a single mutagenic primer set, eliminating a cluster of rare codons in a single experiment. It is likely that this method can support changes of even more than six base pairs as long as the flanking sequences are of sufficient length.

## 4. Conclusion

The COE-PCR method described here combines the strengths of the OE-PCR method with the speed and ease of the AOE-PCR method. It is faster than conventional OE-PCR as it shortens the number of PCR cycles to 40–45 cycles, and it eliminates the need for DNA purification if single bands are obtained in the primary PCR reactions. This method is useful when multiple base pair changes are desired, such as in codon optimization.

## Figures and Tables

**Table 1:** List of primers used for COE-PCR showing restriction sites and base substitutions.

Primer	Sequence (5′ → 3′)	Primer length (bp)	*T*_*m*_ (°C)	Length of overlapping bases (bp)
FGSP1	GAAGTCGCAACTAGTTTCTGACATGGAAAAC	31	60	—
RGSP1	CGAAAAACCCCGGGAAAGGGAGG	23	61	—
FGSP2	GCGGTTCCATGGGTCTAAGG	20	58	—
RGSP2	GGATCCGATATCAGCCATGGTTTTT	25	58	—
FMP1	GCTAAGGTGAT**tc**G**c**CGTGTTATTCC	26	61	26
RMP1	GGAATAACACG**g**C**ga**ATCACCTTAGC	26	61	
FMP2	CCTATGGAGAAACT**g**AT**t**ATTTACCGCTTAAA	32	61.3	32
RMP2	TTTAAGCGGTAAAT**a**AT**c**AGTTTCTCCATAGG	32	61.3	
FMP3	TTGCAATTACCC**g**AT**t**GTCCAACAAATG	28	59.4	28
RMP3	CATTTGTTGGAC**a**AT**c**GGGTAATTGCAA	28	59.4	
FMP4	CTTGTTG**c**G**t**GGGTTCA	17	45.2	17
RMP4	TGAACCC**a**C**g**CAACAAG	17	45.2	
FMP5	TCACTGG**c**G**tc**G**c**TGGGCAG	20	46.5	20
RMP5	CTGCCCA**g**C**ga**C**g**CCAGTGA	20	46.5	
FMP6	CATTGTCCT**g**GAG**c**G**t**CG**c**CTTAAACAA	28	41.9	28
RMP6	TTGTTTAAG**g**CG**a**C**g**CTC**c**AGGACAATG	28	41.9	
FMP7	GAGTAATTTA**c**G**c**ATG**c**G**tc**G**c**AGTGATCTTC	32	<40	32
RMP7	GAAGATCACT**g**C**ga**C**g**CAT**g**C**g**TAAATTACTC	32	<40	
FMP8	GCCCCCTTCTA**c**AT**tagt**TT**t**TATATGAAGTC	32	45	32
RMP8	GACTTCATATA**a**AA**acta**AT**g**TAGAAGGGGGC	32	45	

Underlined are restriction sites. Substituted bases are bold and written in lowercase.

**Table 2:** Details of mutagenesis experiments showing combination of primers in the primer set for the intended base substitutions.

Primer set	Primers	Expected size (bp)	Base pair changes	Joined full length DNA size (bp)	Position of mutation on Stisa2^*∗*^
1	FGSP1 + RMP1	387	AT**a a**G**g**→AT**t c**G**c**	1925	1002–1005
	RGSP1 + FMP1	1564			
2	FGSP1 + RMP2	513	CT**a** AT**a**→CT**g** AT**t**	1925	1125–1128
	RGSP1 + FMP2	1444			
3	FGSP1 + RMP3	931	CC**c **AT**a**→CC**g** AT**t**	1925	1545–1548
	RGSP1 + FMP3	1022			
4	FGSP1 + RMP4	1019	**a**G**a**→**c**G**t**	1925	1639–1641
	RGSP1 + FMP4	923			
5	FGSP1 + RMP5	1166	**a**G**g a**G**a**→**c**G**t c**G**c**	1925	1783–1788
	RGSP1 + FMP5	779			
6	FGSP1 + RMP6	1441	CT**a **GAG** a**G**a **CG**a**→CT**g **GAG** c**G**t **CG**c**	1925	2052–2061
	RGSP1 + FMP6	512			
7	FGSP1 + RMP7	1649	**a**G**a **ATG **a**G**a a**G**a**→**c**G**c **ATG **c**G**t c**G**c**	1925	2257–2268
	RGSP1 + FMP7	308			
8	FGSP2 + RMP8	469	TA**t** AT**c tcc **TT**c**→TA**c** AT**t agt **TT**t**	2685	615–624
	RGSP2 + FMP8	2248			

Substituted bases are bold and written in lowercase.

^*∗*^Position of mutation on *Stisa2* gene sequence (Accession: AY132997).

**Table 3:** Results of primary and secondary PCR reactions with different primer sets.

Primer set	First primary PCR			Second primary PCR			Secondary PCR
	Estimated band size	Optimization required?	Gel excision required?	Estimated band size	Optimization required?	Gel excision required?	Estimated band size
1	400	No	No	1500	No	Yes	1900
2	500	Yes	Yes	1400	No	Yes	1900
3	900	No	No	1000	No	No	1900
4	1000	No	No	900	No	No	1900
5	1100	No	No	800	No	Yes	1900
6	1400	Yes	Yes	500	No	No	1900
7	1600	No	No	300	No	No	1900
8	450	No	No	2250	No	No	2700

## Abbreviations

COE-PCR: Combined overlap extension PCR
OE-PCR: Overlap extension PCR
AOE-PCR: Asymmetrical overlap extension
GSP: Gene specific primers
FGSP: Forward gene specific primer
RGSP: Reverse gene specific primers
FMP: Forward mutagenic primers
RMP: Reverse mutagenic primers
MP: Mutagenic primers
TOP: Trityl-on-purified.
